# Attachment-Related Anxiety and Religiosity as Predictors of Generalized Self-Efficacy and Dispositional Hope

**DOI:** 10.11621/pir.2022.0302

**Published:** 2022-09-15

**Authors:** Emrullah Ecer

**Affiliations:** a Ural Federal University, Ekaterinburg, Russia

**Keywords:** Adult Attachment Styles, Dispositional Hope, Generalized Self-Efficacy (GSE), Focus on Limitation (FOL), Future Time Opportunities (FTO), Experience in Close Relationships-Revised

## Abstract

**Background:**

Attachment-related anxiety and avoidance have a significant impact on self-esteem, optimism, and hope. Moreover, previous studies have shown that religiosity can also be an important factor in promoting hope and emotional regulation.

**Objective:**

The first aim of this study was to explore the relationship between attachment-related anxiety, attachment-related avoidance, dispositional hope, generalized self-efficacy (GSE), future time perspective (FTP) as future time opportunities (FTO), and focus on limitations (FOL). The second purpose was to detect the effect of religiosity on hope, GSE, and FTP.

**Design:**

The study involved 153 Turkish participants (Females n = 81, 52.9%), between the ages of 18 and 66, who filled out the Dispositional Hope Scale, and the Experience in Close Relationship-Revised (ECR-R), FTP, and GSE inventories via a Google survey. A Pearson correlation test, multiple linear regression analysis, and an independent t-test were computed.

**Results:**

Attachment-related anxiety was inversely related to dispositional hope and GSE, while it was positively associated with FOL. Attachment-related avoidance was negatively related to dispositional hope and FTO, whereas it was positively correlated with FOL. FTO was positively correlated with GSE and dispositional hope. Religious participants had a higher level of dispositional hope and GSE than non-religious participants. A lower level of attachment-related anxiety and religiosity was associated with a higher level of GSE and hope. Females showed a higher level of makeup agency than men, whereas the men reported a higher level of FOL than the women.

**Conclusion:**

This study concluded that both attachment-related anxiety and religiosity were predictors of hope and GSE. Gender differences played a significant role in FOL and make-up agency. Moreover, it was found that attachment-related anxiety and avoidance have different functions in GSE and FTO.

## Introduction

Attachment theory underlines the importance of the relationship between mothers and children in children’s development (Ainsworth et al., 1978). The quality of the interaction between mothers and children shapes children’s cognitive skills (O’Connor & McCartney, 2007), emotional regulation (Brumariu, 2015), and attention skills (Clarke et al., 2002). The skills learned during childhood play an important role in a romantic relationship. Some studies reveal that the quality of attachment figures in childhood shapes attachment styles in adulthood (Hasim et al., 2018). For instance, early caregiver experiences have a significant impact on the quality of a person’s intimate relationships as an adult (Ainsworth et al., 2015; Mikulincer & Shaver, 2016). Nevertheless, gender differences play a significant role in the parents’ impact on romantic relationships. If males are insecurely attached to their fathers, they are more likely to report a higher level of attachment-related anxiety and avoidance in their romantic relationships. On the flip side, if females have an insecure relationship with their mothers, they are more vulnerable to aggressive behavior (Santona et al., 2019).

Furthermore, attachment styles in childhood shape brain development in adulthood. A longitudinal study has examined the brain activity of 59 babies 18 months of age 20 years later. The research found that babies who had an insecure attachment experience at 18 months, had a wider amygdala at 22 years old than babies with a secure attachment experience (Moutsiana et al., 2015).

The continuous scale of adult attachment styles is divided into two dimensions-attachment-related anxiety and avoidance. There is a further classification within the attachment model by Griffin and Bartholomew (1994) which I utilized in this study. Participants with a higher level of attachment-related anxiety and avoidance fall into the fearful group. A higher level of attachment-related anxiety and a lower level of attachment-related avoidance refers to a preoccupied attachment style, whereas a higher level of attachment-related avoidance and a lower level of anxiety indicates a dismissive attachment style. If individuals report a lower level of attachment-related anxiety and avoidance, they are classified as belonging to the securely attached group. However, since measurements on the continuous scale of attachment-related anxiety and avoidance are more reliable (Fraley et al., 2015), I preferred to use the two-dimensional models of attachment styles as attachment-related anxiety and avoidance. However, to understand the concept of attachment styles on other variables, they are further classified into groups as well.

People’s attachment styles adjust their social interactions. Securely attached folks are better at having social interactions (Shaver & Mikulincer, 2006). Nevertheless, a high level of attachment-related avoidance and anxiety distorts personal skills. Those people are vulnerable to psychological problems and report a lower level of dispositional mindfulness. It should be noted that participants with a higher level of attachment-related anxiety exhibit behavior that is inversely associated with mindfulness, accepting emotions, quality of life, and self-acceptance than participants with a higher level of attachment-related avoidance (Calvo et al., 2020). Therefore, we can conclude that attachment-related anxiety and avoidance have different effects on psychological dimensions.

There are slight differences in psychological well-being across attachment styles. Both attachment-related avoidance and anxiety are negatively related to psychological well-being. Promoting a high level of self-esteem may elevate the level of mental wellbeing, personality traits, and secure attachment style (Marrero-Quevedo et al., 2019). Therefore, it should be highlighted that self-esteem may control attachment styles.

### Hope

Negative interactions with attachment figures affect people’s thoughts, perceptions, emotional regulations, and hope. It has been found that people with a secure attachment style are prone to trust other people. Securely attached participants with a high level of hope are less likely to suffer from burnout syndrome (Simmons et al., 2009). In addition to that, individuals with a higher level of attachment-related avoidance are likely to feel hopeless (Gnilka et al., 2013).

Hope has two elements. The first one is called the make-up pathway, which is related to anticipation of the future, goal-oriented thoughts, and the perception of self-ability. The second one is the agency thoughts that are relevant to a motivation-orientation and desire to reach certain goals (Snyder, 2000). Hope is interwoven with optimism. Inner and external factors adjust the perception of hope. Hope elevates emotional regulation, psychological well-being (Peh et al., 2016), mindfulness (Munoz et al., 2018), and life satisfaction (Kardas et al., 2019). Hence, it can be specified that the expression of emotions, mental well-being, and thinking patterns are associated with hope.

Hope controls the relationship between psychological disorders and attachment styles. A previous study found that insecurely attached people are vulnerable to psychological disorders such as depression, general anxiety disorder, social anxiety, and eating problems. When people report a higher level of hope, these problems are alleviated (McDermott et al., 2015).

Thus, it should be underlined that hope and insecure attachment styles are inversely correlated. In addition, hope can regulate the negative effects of psychological diseases if people have attachment difficulties. Even though people who are exposed to negative life events may develop symptoms of depression, hope decreases these problems (Visser et al., 2013). Thus, it should be said that hope and attachment styles are related to each other. Having a secure attachment style boosts hope and ego resilience, which results in psychological well-being.

### Generalized Self-Efficacy (GSE)

Self-efficacy refers to a person’s motivation, goals, and sense of capability (Bandura, 1977). Generalized self-efficacy (GSE) accounts for the capability to find an adaptive solution for unexpected events and/or stressful conditions (Schwarzer, 1994). It has been found that GSE is negatively correlated with depression symptoms, anxiety, and helplessness (Behice, 2006).

A review of the literature has suggested that securely attached children tend to explore the world. They are independent and have a higher level of self-esteem compared to insecurely attached children. As those children grow up, they have a higher chance of becoming resilient and competent. On the other hand, insecurely attached children’s self-perception deteriorates. They report more impulsivity. They indicate a higher problem in their social relationships. They are less trustful and skilful compared with securely attached children (Hong & Park, 2012).

The same effect is observed in adulthood. People with a higher level of attachment-related anxiety and avoidance report a lower level of self-efficacy. Having a lower level of attachment-related anxiety and avoidance increases the level of self-efficacy. Those people with a lower level of attachment-related anxiety and avoidance with a higher level of self-efficacy show a higher level of mental well-being (Bender & Ingram, 2018). In addition to attachment styles, hope has a significant impact on self-efficacy. Hope leads people to have a higher level of self-efficacy (O’Sullivan, 2011).

If individuals are hopeful with a high level of GSE, they are more likely to have an optimistic view of the future. It has been suggested that a future time perspective is positively related to GSE. When people have a higher level of self-efficacy, they show an optimistic orientation toward the future (Zebardast et al., 2011). Thus, it should be concluded that hope, and a lower level of attachment-related anxiety and avoidance, promote GSE.

### Future Time Perspective (FTP)

Future time perspective (FTP) theory examines a person’s expectations about the future, goals, and perception of the world (Lang & Cartensen, 2002). A previous study has found that when people had a higher level of FTP, they were more likely to have a higher level of consciousness and self-esteem (Akirmak, 2019). The FTP is a cognitive process that shapes how people evaluate their lives. In this case, it has similar features to hope. Hope affects both FTP and life satisfaction (Dwivedi & Rastogi, 2016).

It can be said that hope can control FTP and induce psychological well-being. If people perform at a higher level of FTP, their levels of aggression, depression, and anxiety decrease. Furthermore, those people with a higher level of FTP can manage their impulsivity (Zimbardo & Boyd, 1999). Hence, it can be determined that FTP plays an important role in regulating emotions and cognitive behavior.

Attachment styles play an important role in emotional and cognitive functions. Attachment styles during childhood are vitally important in determining which strategies children will have in the future (Laghi et al., 2009). Hence, it can be said that attachment styles in adulthood will have a significant effect on FTP.

### Religiosity

Attachment styles are related to religiosity values. One study has reported that people who adhered to religious traditions tended to have an avoidant attachment style, while the anxious attachment style was not related to religious rituals (Cobb, 2017).

Religiosity plays a crucial role in creating mental well-being and cognitive skills. Religious people are more hopeful than non-religious individuals (Hasson-Ohayon et al., 2009). The positive effect of religiosity is observed in their level of happiness. Religious participants have been found to be more likely to have a higher level of happiness than non-religious ones (Abdel-Khalek & Lester, 2017).

This result could be due to the fact that religious people are integrated into a group. As individuals engage in church activities, their life satisfaction increases (Leondari & Gialamas, 2009). The integration with the group helps religious individuals to have good coping strategies for their emotional regulation (Vishkin et al., 2016). When religious people are faced with stressful events, they are better at finding new coping strategies to deal with them than non–religious people (Agbaria, 2021). Therefore, those cognitive and emotional advances may aid in helping for religious people to have higher levels of hope and GSE.

In conclusion, this study had two aims. The first was to explore the relationship between attachment-related avoidance and anxiety with dispositional hope, GSE, and FTP. The second was to analyze the effect of religiosity on hope, GSE, and FTP.

There were two research questions:

1. What is the relationship between dispositional hope, GSE, FTP, and attachment styles?
2. Does religiosity influence the level of hope, GSE, and FTP?

## Methods

### Participants and Design

The study was carried out between December 21, 2020 and January 31, 2021 in Turkey. Participants were contacted through social media portals. There were 153 Turkish participants (Females *N* = 81, 52.9%). The mean age of the participants was 34.76 (*SD* = 13; range 18-66 years). The majority of participants reported that “I am religious” (n = 103, 67.3%). The majority of the participants had secure and preoccupied attachment styles (Secure n = 57, 37.3%; Preoccupied n = 57, 37.3%). Most of the participants had a university degree (n = 111, 72.5%). Detailed information on the sample is shown in *Table 1.*

**Table 1:** Demographic Variables

Variables	Frequency	Percentage
	*N*	*(%)*
Gender		
Female	81	52.9
Male	72	47.1
Education		
Primary	3	2
High school	19	12.4
Bachelor	111	72.5
Master	17	11.1
PH.D.	3	2
Religiosity Status		
“I am religious”	103	67.3
“I am not religious”	50	32.7
Attachment Styles		
Secure	57	37.3
Fearful	57	37.3
Preoccupied	20	13.1
Dismissive	19	12.4

The participants filled out the Dispositional Hope Scale, the Generalized Self-efficacy Scale, the Future Time Perspective scale, and the Experience in Close Relationships scale via a Google form. Individuals did not gain any benefit by participating in the study. Participants had to be older than 18 years old to join. Eight participants were excluded due to their extreme outlier scores.

### Procedure

#### Dispositional Hope Scale

The Dispositional Hope Scale (Snyder et al., 1991) measures how people find ways to cope with stress, to what extent they are goal-oriented, and how they handle problems. The scale has three time perspectives: past, future, and present. The hope scale has two components: 1) the make-up of the person’s agency (goal-directed energy) and 2) the person’s make-up pathway to organize strategies toward goals. In the current study, the Turkish version of the dispositional hope scale (Tarhan & Bacanli, 2015) was used. The make-up of agency sub-scale had four statements ranging from the past to current times regarding the perception of accomplishment (*e.g.*, “I have been pretty successful in life”). The make-up pathway sub-scale had four statements consisting of descriptions about handling problems in difficult situations (*e.g.*, “I can think of many ways to get out of a jam”). The scale ranged from “1 = Definitely False” to “8 = Definitely True.” Higher scores indicated a higher level of hope.

In the current study, the dispositional hope scale showed a good internal consistency reliability score (McDonald Omega’s coefficient = .81). Both of the two subscales showed an acceptable reliability score, and the internal reliability score for the make-up pathway sub-scale (McDonald Omega’s coefficient = .76), and for the makeup agency sub-scale (McDonald Omega’s coefficient = .75).

#### Generalized Self-Efficacy Scale (GSE)

The generalized self-efficacy scale (GSE) (Jerusalem & Schwarzer, 1995) analyzes the perception of self-efficacy in terms of solving problems in the face of unexpected issues. It includes 10 statements including self-beliefs, strategies, and optimism (*e.g.,* “If I am in trouble, I can usually think of a solution”). In the current study, the Turkish version of the GSE (Erci, 2005) was used. The scale ranged from “1= Not at all True” to “4 = Exactly True.” The scale had a good internal reliability score (McDonald Omega’s coefficient = .89).

#### Future Time Perspective Scale (FTP)

The Future Time Perspective scale (Cartensen & Lang, 1996) emphasizes people’s perceptions and goals towards their prospects in the future. The scale consists of 10 statements about people’s thoughts, possibilities, and expectations regarding their future. The scale is divided into two categories: 1) future time opportunities (FTO) with seven statements (*e.g.,* “I expect that I will set many new goals in the future”), and 2) focus on limitations (FOL), which includes three descriptions regarding the perception of limited prospects the future (*e.g.,* “There are only limited possibilities in my future”).

The Turkish version of the FTP was used (Ozekes &, Soylu, 2019). The scale ranged from “1= Very Untrue” to “7 = Very True.” The FTO sub-scale had an excellent reliability score (McDonald’s Omega’s coefficient = .90), while FOL had a high internal reliability score (McDonald’s Omega’s coefficient = .83).

#### The Experience in Close Relationships - Revised Questionnaire (ECR-R)

The revised version of the Experience in Close Relationships scale (Fraley et al., 2000) was used to determine the anxious and avoidant attachment styles in the adult population. The questionnaire includes 36 questions exploring attachment-related anxiety and avoidance. The attachment-related anxiety scale measures to what extent a person is sensitive to abandonment, fear of being unloved, and the level of trust in the romantic relationship (*e.g.,* “I often worry that my partner doesn’t really love me”).

The attachment-related avoidance sub-scale is defined as having a close, intimate, safe, comfortable relationship with partners, and the quality of communication between partners *(e.g.,* “I find it easy to depend on romantic partners”). The Turkish version of the Experience in Close Relationships scale (Selçuk et al., 2005) was used in the current study. The scale ranged from “1 = Strongly Disagree” to “7 = Strongly Agree.” Both of the sub-scales showed high internal consistency scores. For anxious attachment, the reliability score (McDonald’s Omega’s coefficient) was .85); for the avoidance attachment, that coefficient was also .85.

### Data Analysis

The data was computed by SPSS, version 26. Skewness, kurtosis, and standardized scores of skewness and kurtosis were measured to determine whether the study was normally distributed.

Z-scores between -3.29 and +3.29 in the moderate sample between 50 <n< 300 qualify results as parametric (Kim, 2013). In this study, the z-scores of all of the subscales ranged between -3.29 and +3.29. Pearson’s correlation test was used to analyze the relationship between dispositional hope, FTP, GSE, and adult attachment styles. An independent t-test was computed to measure the effects of gender and religiosity on FTP, GSE, and dispositional hope. Religiosity, attachment styles, and gender were chosen as independent variables, while the dispositional hope scale, GSE, and FTP were dependent variables.

Moreover, the classification of attachment styles as secure, preoccupied, fearful, and dismissive was measured by the median of attachment-related anxiety and avoidance. In this case, the effect of attachment styles on GSE, FTP, and hope was explored using One-Way ANOVA.

Multiple linear regression analyses were computed to explore the effects of religiosity and attachment styles on FTP, GSE, and hope. The internal consistency reliability scores of the scales were computed using the method of McDonald’s Omega coefficient analysis (Flora, 2020).

In addition, G*Power (Faul et al., 2020) was used to examine whether the study had a large enough sample power size. The type of the power was the bivariate correlation model of the post hoc two tails for Pearson’s correlation test, while in the fixed model, R2 deviation from zero was measured to reveal the multiple linear regression sample size.

**Table 2:** Descriptive Statistics

Measure	Mean	SD	Kurtosis (SE)	Skewness (SE)	Kurtosis (Z)	Skewness (Z)
FOL	13.5	5.10	.80, (.39)	–.32, (.20)	2.05	–1.6
FTO	33.9	9.27	–.08, (.39)	–.52, (.20)	–.20	–2.60
Hope	51.8	7.32	–.36, (.39)	–.48, (.20)	–.92	–2.40
Make up	27.2	3.97	–.50, (.39)	–.63, (.20)	–1.28	–3.15
Agency	24.6	4.45	–.02, (.39)	–.61, (.20)	–.051	–3.05
Anxious	67.1	18.47	.28, (.39)	.48, (.20)	0.72	2.40
Avoidant	54.3	17.50	–.36 (.39)	14, (.20)	–.92	–.70
GSE	30.0	5.63	.11, (.39)	–.26,(.20)	.28	–1.30

*Note. SD = Standard Deviation, SE= Standard Error, Z= Standard Score*

## Results

The results indicated that dispositional hope was positively related to FTO (r = .45, p = .0001) and GSE (r = .73, p = .0001), while it was negatively correlated with attachment-related anxiety (r = –.22, p = 0.006) and avoidance (r = –.19, p = .02).

FOL was positively correlated with attachment-related anxiety (r = .31, p = .0001) and avoidance (r = .29, p = .0001), while it was negatively related to FTO (r = –.35, p = .0001). FTO was negatively correlated with attachment-related avoidance (r = –.17, p = .04), whereas it was positively associated with GSE (r = .41, p = .0001).

GSE was negatively associated with attachment-related anxiety (r = –.29, p = .0001) and attachment-related anxiety was positively correlated with attachment-related avoidance (r = .35, p = .0001).

**Table 3:** Pearson’s Correlation Test between Variables

Variable	1	2	3	4	5	6	7	8
1. FOL	–							
2. FTO	–.35***	–						
3. Hope	–.11	.45***	–					
4. Makeup	–.05	.40***	.85***	–				
5. Agency	–.14	.38***	.88***	.51***	–			
6. Anxious	.31***	–.06	–.22**	–.21**	–.18*	–		
7. Avoidant	.29***	–.17*	–.19*	–.15	–.18*	.35***	–	
8. GSE	–.07	.41***	.73***	.65***	.62***	–.29***	–.14	–

Note. FOL = Focus on Limitations, FTO = Future Time Opportunities, Hope = Dispositional Hope, Makeup = The make up the Pathway, Agency = Make up the Agency, GSE = Generalized Self-Efficacy. * p < .05, ** p < .01, *** p < *.001*

One-Way ANOVA showed that attachment styles had a significant influence on hope: F (3, 149) = 4.09, p = .008. Participants with a fearful attachment style had a lower level of hope (*M* = 49.26, *SD* = 7.70) than individuals with a dismissive attachment style (*M* = 52.00, *SD* = 6.59), preoccupied attachment style (*M*=52.85, *SD* = 6.13), or secure attachment style (*M* = 53.81, *SD* = 6.95).

Attachment styles had a significant impact on GSE: F (3, 149) = 3.59, p = .015. People with secure attachment styles had a higher level of GSE (*M* = 31.77, *SD* = 5.60) than individuals with dismissive attachment styles (*M* = 29.95, *SD* = 4.41), preoccupied attachment styles (*M* = 29.30, *SD* = 4.82, or fearful attachment styles (*M* = 28.46, *SD* = 5.88).

Attachment styles had a significant effect on FTO: F (3, 149) = 4.29, p = .006. Participants with a fearful attachment style had a lower level of FTO (*M* =30.75, *SD* = 9.09) than securely attached individuals (*M* = 34.79, *SD* = 9.73), or those with a preoccupied attachment style (*M* =37.90, *SD* = 7.90) or a dismissive attachment style (*M* = 36.32, *SD* = 7.13).

Attachment styles had a significant influence on FOL: F (3,149) = 6.03, p = .001. Participants with a fearful attachment style showed a higher level of FOL (*M* = 15.26, *SD* = 4.54) than those with a dismissive attachment style (*M* = 14.74, *SD* = 4.17), preoccupied attachment style (*M* = 13.05, *SD* = 5.07), or a secure attachment style (*M* = 11.54, *SD* = 5.30).

The results suggested that religious people significantly demonstrate a higher level of hope than non-religious individuals. The hope score for people who were religious was *M* = 53.00, *SD* = 7.00), as opposed to the hope score for non-religious participants (*M* = 50.00, *SD* = 7.70); conditions: t (159) =2.20, p = .02).

Moreover, religious people showed a higher level of GSE than non-religious individuals. The GSE scores for religious people were *M* = 31.00, *SD* = 5.30, while those for non-religious participants were *M* = 28.50, *SD* = 7.70; conditions; t (151) = 2.20, p = .03.

The results indicate that women have a significantly higher level of make-up agency than men. The mean score for women was *M* = 25.3, *SD* = 4.09, while that for men was *M* = 23.8, *SD* = 4.73); conditions; t (151) = 2.07, p = .04).

In addition, the men had a higher level of FOL than the women. The mean of FOL for women was *M* = 12.59, *SD* = 5.09; the mean of FOL for men was *M* = 14.57, *SD* = 4.94; conditions; t (151) = –.2.43, p = .016).

**Table 4:** Multiple regression analysis measured the effects of attachment-related anxiety and irreligiosity on GSE

	Unstandardized Coefficients		P	95.0% CI	
	Estimate	SE		LL	UL
Anxious	–.09	.02	.0001	–.14	–.04
Irreligiosity	–2.22	.91	.016	–4.03	–.41

*Note. DV = GSE, R2=.12, Adj. R2 = .11, CI = Confidence Interval, LL = Lower Limit, UL=Upper Li*

The first model of the multiple regression analysis data showed that both irreligiosity and attachment-related anxiety are related to GSE: F (2, 150) = 10.36, p = .0001, R2 = .12. Both attachment-related anxiety (B = –.09, p = .0001) and irreligiosity (B = –.2.22, p = 0.16) are negatively correlated with GSE.

The second model of the multiple linear regression analysis showed that both irreligiosity and attachment-related anxiety are associated with hope; F (2, 150) = 6.78, p = .002, R2 = .08). Both attachment-related anxiety (B = –.09, p = .005) and irreligiosity (B = –2.87, p = 0.19) were negative predictors of hope.

**Table 5:** Multiple regression analysis measured the effects of attachment-related anxiety and irreligiosity on dispositional hope

	Unstandardized Coefficients		P	95.0% CI	
	Estimate	SE		LL	UL
Anxious	–.09	.03	.005	–.15	–.03
Irreligiosity	–2.9	1.2	.02	–5.3	–.47

*Note. DV = Dispositional Hope, R2=.08, Adj. R2=.07, CI=Confidence Interval, LL=Lower Limit, UL=Upper Limit*

**Table 6:** Multiple regression analysis measured the effects of attachment-related anxiety and avoidance on FOL

	Unstandardized Coefficients		P	95.0% CI	
	Estimate	SE		LL	UL
Anxious	.07	.22	.004	.02	.11
Avoidant	.06	.24	.012	.01	.11

*Note. DV = FTL, R2 = .13, Adj. R2 = .12, CI = Confidence Interval, LL = Lower Limit, UL=Upper Limit*

The third model of the multiple linear regression analysis found that attachment-related avoidance and anxiety were positively associated with FOL: F (2,150) = 11.5, p = .0001, R2 = .13. Attachment-related avoidance (B = .06, p = .01) and anxiety (B = .07, p = .004) were positively related to FOL.

## Discussion

This study had two main objectives. The first one was to detect the relationship between attachment-related dimensions, hope, GSE, and FTP. The second one was to analyze the effects of religiosity on FTP, GSE, and hope.

Previous findings indicated that when individuals have a higher level of hope, they tend to have a higher score on GSE (Feldman & Kubota, 2015). Both self-efficacy and hope promote mental health (Liu et al., 2018). In addition, a previous study suggested that GSE and hope are strongly related to each other (r = .98) and are inhibitors of a higher level of depression (D’Souza et al., 2020). Moreover, hope adjusts FTP (Adelabu, 2008). The current study found that hope and GSE are strongly related to each other (r = .73), and both are associated with FTO, while they are not correlated with FOL. Hence, it should be highlighted that both hope and GSE might promote a positive orientation toward the future.

Another variable that is related to mental well-being and hope is religiosity. Being religious has a positive influence on hope and mental health (Al Eid et al., 2021). This study found that religiosity has a significant impact on GSE and hope. When participants report that they are religious, they are more likely to have a higher level of hope and GSE than non-religious participants.

On the other hand, having a higher level of attachment-related anxiety and avoidance distorts hope (Shorey et al., 2003). Nevertheless, attachment to God plays an important role in the regulation of hope and self-esteem (Ren, 2020). When people are religious, their level of hope increases (Pahlevan Sharif et al., 2021). Not just attachment to God, but also adult attachment styles regulate self-efficacy (Bender & Ingram, 2018). Securely attached participants exhibited a higher level of hope than insecurely attached individuals (Demirtaş, 2019). Attachment-related anxiety was inversely related to hope (Blake & Norton, 2014). In the current study, both attachment-related anxiety and irreligiosity were inversely related to GSE and hope. It should be noted that those variables affect hope more than GSE does.

A systematic review found that mental well-being and goal-oriented behaviors were related to FTP (Kooij et al., 2018). In the current study, FTP was divided into FTO and FOL. FTO is a protective element against depression symptoms, while FOL is related to the stress system. However, FOL is not associated with subjective mental health (Kozik et al., 2015). A study on a Turkish sample showed that FOL was negatively associated with psychological and physical health (Soylu & Ozekes, 2019).

Furthermore, since FTP refers to a thinking orientation toward the future. it is related to the individual’s self-perception. Individuals with a high level of self-efficacy have a higher level of FTP (Dutt & Wahl, 2019).

Moreover, a secure attachment style controls GSE (Bender & Ingram, 2018). Therefore, it can be hypothesized that attachment-related anxiety and avoidance are negatively associated with GSE and FTP. Nevertheless, in this case, the role of attachment-related avoidance should be highlighted. Individuals with a higher level of attachment-related avoidance are autonomous and self-reliant. They might feel better when they are alone (Wardecker et al., 2020). In this case, the current study revealed that attachment-related anxiety was negatively associated with dispositional hope and GSE, and it was positively correlated with FOL. However, attachment-related anxiety was not significantly correlated with FTO. On the other hand, attachment-related avoidance was negatively related to hope and FTO but positively correlated with FOL. However, attachment-related avoidance was not significantly associated with GSE. Thus, it can be concluded that attachment-related anxiety and avoidance have different functions on GSE and FTO. Attachment-related avoidance is a style related to FTO, whereas attachment-related anxiety is associated with GSE.

When attachment styles were classified into secure, dismissive, preoccupied, and fearful groups, we found that participants with fearful attachment styles were prone to score lower on hope, GSE, FTO, and higher score on FOL. Having a secure relationship promotes a higher level of hope, GSE, and a lower level of FOL. Nevertheless, participants with dismissive and preoccupied attachment styles were more likely to have a higher level of FTO that should be detected in the future.

A previous study showed that the level of hope was higher in men than in women (Naik & Yadav, 2017). Furthermore, a recent study after the pandemic suggested that women were more vulnerable to generalized anxiety disorder and stress (Chima et al., 2022). However, the current study indicated that males were more likely to feel that their future was limited and were less likely to engage in goal-directed behavior than the female participants. Therefore, cultural factors should be taken into account in examining the effects of gender.

## Conclusion

This study has several conclusions. The first is that attachment-related anxiety is positively associated with FOL and inversely correlated with dispositional hope and GSE. Attachment-related avoidance is positively related to FOL and negatively associated with dispositional hope and FTO. Attachment-related avoidance is not significantly associated with GSE and the make-up pathway. Dispositional hope is positively related to FTO and GSE, while it is not significantly correlated with FOL. GSE is positively correlated with FTO, while it is not correlated with FOL.

The second major conclusion is that religiosity has a significant impact on hope and GSE. Religious individuals have a higher level of dispositional hope and GSE than non-religious people.

The third finding is that women perform a higher level of makeup agency than men, whereas men have a higher level of FOL than women.

The fourth is that both irreligious and attachment-related anxiety are negatively predicted to be related to GSE and dispositional hope.

The fift h is that attachment-related anxiety and avoidance are positively predicted to be related to FOL.

Sixth, when attachment styles are divided into categories, it can be concluded that having a secure attachment style is advantageous for hope, GSE, FOL, and dispositional hope, whereas a fearful attachment style impairs those skills. It should be noted that individuals with a dismissive and preoccupied attachment style have a higher level of hope than securely attached participants, which should be considered in the future.

## Limitations

The present study had several limitations. First, the levels of hope, GSE, FTP, and attachment styles might have been sensitive to the outbreak of COVID. For instance, it was found that the level of attachment-related anxiety and avoidance was higher than in the study conducted in 2019 in Turkey (Koç et al., 2019). Previous studies demonstrated that the mental well-being of people deteriorated during the COVID-19 pandemic, and that age is a significant predictor of mental well-being (O’Connor, et al., 2020).

Second, the study did not focus on any specific age group, and age differences might be an important factor in these scores. Third, the study was based on a Turkish sample; it would be interesting to generalize these scores to other cultures. The fourth limitation was the sample size. The correlation between attachment-related anxiety and FTO (.56) and the relationship between attachment-related avoidance and dispositional hope (.66) had a lower sample size than 80%. Moreover, the study was a cohort study so that the results indicated the relationship between variables rather than determining causal effects.

A specific religious behavioral scale might be used in the future rather than simply asking the binary question, Are you religious or not? Nevertheless, the present study was the first study to examine the relationship between religiosity, FTP, attachment styles, and dispositional hope in a Turkish sample.
